# Maternal BMI Associations with Maternal and Cord Blood Vitamin D Levels in a North American Subset of Hyperglycemia and Adverse Pregnancy Outcome (HAPO) Study Participants

**DOI:** 10.1371/journal.pone.0150221

**Published:** 2016-03-04

**Authors:** Jami L. Josefson, Anna Reisetter, Denise M. Scholtens, Heather E. Price, Boyd E. Metzger, Craig B. Langman, HAPO Study Cooperative Research Group

**Affiliations:** 1 Ann & Robert H. Lurie Children’s Hospital of Chicago, Division of Endocrinology, Chicago, Illinois, United States of America; 2 Department of Pediatrics, Northwestern University Feinberg School of Medicine, Chicago, Illinois, United States of America; 3 Department of Preventive Medicine, Northwestern University Feinberg School of Medicine, Chicago, Illinois, United States of America; 4 Ann & Robert H. Lurie Children’s Hospital of Chicago, Division of Kidney Diseases, Chicago, Illinois, United States of America; 5 Department of Medicine-Endocrinology, Northwestern University Feinberg School of Medicine, Chicago, Illinois, United States of America; College of Tropical Agriculture and Human Resources, University of Hawaii, UNITED STATES

## Abstract

**Objective:**

Obesity in pregnancy may be associated with reduced placental transfer of 25-hydroxyvitamin D (25-OHD). The objective of this study was to examine associations between maternal BMI and maternal and cord blood levels of 25-OHD in full term neonates born to a single racial cohort residing at similar latitude. Secondary objectives were to examine associations between maternal glucose tolerance with maternal levels of 25-OHD and the relationship between cord blood 25-OHD levels and neonatal size.

**Methods:**

This study was conducted among participants of the Hyperglycemia and Adverse Pregnancy Outcomes (HAPO) Study meeting the following criteria: residing at latitudes 41–43°, maternal white race, and gestational age 39–41 weeks. Healthy pregnant women underwent measures of height, weight, and a 75-g fasting oral glucose tolerance test (OGTT) at approximately 28 weeks gestation. Maternal and cord blood sera were analyzed for total 25-OHD by HPLC tandem mass spectrometry. Statistical analyses included ANOVA and linear regression models.

**Results:**

Maternal and cord blood (N = 360) mean levels (sd) of 25-OHD were 37.2 (11.2) and 23.4 (9.2) ng/ml, respectively, and these levels were significantly different among the 3 field centers (ANOVA p< 0.001). Maternal serum 25-OHD was lower by 0.40 ng/ml for BMI higher by 1 kg/m^2^ (p<0.001) in an adjusted model. Maternal fasting plasma glucose, insulin sensitivity, and presence of GDM were not associated with maternal serum 25-OHD level when adjusted for maternal BMI. Cord blood 25-OHD was lower by 0.26 ng/ml for maternal BMI higher by 1 kg/m^2^ (p<0.004). With adjustment for maternal age, field center, birth season and maternal serum 25-OHD, the association of cord blood 25-OHD with maternal BMI was attenuated. Neither birth weight nor neonatal adiposity was significantly associated with cord blood 25-OHD levels.

**Conclusion:**

These results suggest that maternal levels of 25-OHD are associated with maternal BMI. The results also suggest that interpretation of neonatal 25-OHD levels may need to incorporate specific maternal factors in addition to season of birth and latitude.

## Introduction

Overweight and obesity have become increasingly common among pregnant women in higher-income countries [1, 2]. The perinatal risks associated with obesity in pregnancy include, in part, operative delivery, gestational diabetes mellitus (GDM), pregnancy induced hypertension, and large-for-gestational age neonates [3, 4]. In addition, there are long-term health complications for the offspring of obese women, as such offspring born to mothers with GDM are more likely to become obese themselves [5] and have reduced insulin sensitivity [6–8], thereby increasing their risk of developing type 2 diabetes mellitus. Vitamin D deficiency is common in obesity [9] and may pose as an additional perinatal and childhood risk among obese pregnant women and their neonates. For example, it has been shown that children born to vitamin D deficient mothers have increased adiposity [10] and higher risk of food allergy [11] in childhood.

Our previous work suggested that obesity in pregnancy was associated with reduced placental transfer of 25-hydroxyvitamin D (25-OHD) [12]. Obese individuals are known to have reduced bioavailability of 25-OHD [9] and this reduction in serum 25-OHD levels places obese pregnant women at higher risk of 25-OHD deficiency. Frank maternal vitamin D deficiency, using levels ranging from less than 12–15.5 ng/ml, has been associated with low birth weight [13], increased rates of small for gestational age births [13, 14], and GDM [15]. However, previous studies on birth weight outcomes and risk of GDM among vitamin D deficient mothers did not adequately address rates of maternal obesity, used an inaccurate vitamin D assay, or studied neonates of varying gestational ages, thus confounding interpretation of results.

We designed the current study to strengthen our hypothesis that obesity in pregnancy is associated with reduced levels of cord blood 25-OHD, by studying full term neonates born to a single racial cohort residing at similar latitude. Additionally, we examined associations between maternal glucose tolerance with maternal levels of 25-OHD and evaluated the relationships between cord blood 25-OHD levels and neonatal weight and adiposity.

## Materials and Methods

This study was conducted among participants enrolled in the Hyperglycemia and Adverse Pregnancy Outcomes (HAPO) Study from the following field centers: Chicago, IL, USA, Cleveland, OH, USA, and Toronto, ON, Canada. HAPO was an epidemiological study designed to investigate associations of non-diabetic glycemic levels with adverse pregnancy outcomes [16]. Following an overnight 8–10 hour fast, healthy pregnant women underwent measures of height, weight, and a 75-g oral glucose tolerance test (OGTT) at approximately 28 weeks gestation. Cord blood was collected at delivery. The institutional review board at each field center (Northwestern University IRB in Chicago, MetroHealth System IRB in Cleveland and Sunnybrook Health Sciences Centre Research Ethics Board in Toronto) approved the HAPO Study, and participants provided written, informed consent to participate.

The analysis presented here was limited to participants meeting the following criteria: residing at latitudes 41–43°, maternal white race, and gestational age 39–41 weeks. Mother/neonate pairs (N = 360) were randomly sampled with evenly distributed seasons of birth for 25-OHD measurements on the relevant blood sample. Maternal BMI categories (kg/m^2^) based upon measurements taken at the OGTT study visit were as follows: normal range 22.6–28.4, overweight 28.5–32.9, and obese >33. These discrete BMI categories were aligned with the standard pre-pregnancy BMI categories of normal range 18.5–24.9, overweight 25.0–29.9, and obese >30 based on a regression of BMI at the time of the HAPO study visit on pre-pregnancy BMI and gestational age at the study visit [17].

Maternal and cord blood sera were analyzed for total 25-OHD by HPLC tandem mass spectrometry as described [18]. A Micromass Quattro Micro triple-quadruple mass spectrometer equipped with a Z-spray ion source and a Waters 2795 Alliance HT HPLC system (Waters Chromatography, Milford, MA) was used. The tandem mass spectrometer is operated in the electrospray positive ionization mode. System operation and data processing are controlled by MassLynx NT 4.0 software (Waters Chromatography). The method was validated previously with the liquid chromatography, tandem mass spectrometry method commercially offered by Mayo Medical Laboratories (Rochester, MN), with the correlation demonstrating a good agreement over a wide range of concentrations. The detection limit was 3.0 ng/ml, and the inter- and intraassay coefficients of variation were below 7.7% across the analytical measurable range. Maternal and cord blood sera were assayed for 25-OHD in one batch to eliminate interassay variation.

Maternal plasma glucose and C-peptide levels were previously analyzed at the central laboratory of the HAPO study [16], as described [19]. Briefly, the Central Laboratory used a “Vitros 750” analyzer for glucose analysis (oxidase/peroxidase method); calibration was verified against plasma samples previously measured by the hexokinase reference method at Ortho-Clinical Diagnostics Company headquarters (Rochester, New York). Serum C-peptide was assayed in 96-well plates on an Autodelfia instrument supplied by Perkin-Elmer. This is a solid phase, two-site fluoro-immunometric assay based on the direct sandwich technique in which two monoclonal antibodies are directed against separate antigenic determinants on the C-peptide molecule. The assay is calibrated against a synthetic C-peptide preparation.

The maternal insulin sensitivity estimate, IS_OGTT C-pep_ for each participant was calculated using the formula determined by Radaelli et al. [20]. This formula incorporates maternal glucose and C-peptide at 0 and 60 minutes of the OGTT.

Neonatal measurements were obtained in a standardized method by trained personnel within 72 hours of delivery as previously described [21]. The anthropometric measurements including weight, length, and skin fold thickness at the flank were obtained in duplicate and the results averaged. A third measurement was taken when required by the protocol [21]. Birth weight was obtained without diaper using a calibrated electronic scale. Length was measured on a standardized plastic length board constructed for use in the HAPO Study. Flank skin fold thickness was measured with calipers (Harpenden, Baty, U.K.) on the neonate’s left side just above the iliac crest on a diagonal fold on the mid-axillary line. Fat mass was calculated from birth weight, length, and flank skin fold according to the equation given by Catalano et al. [22] and percent body fat was then calculated (fat mass/birth weight * 100).

After visual inspection for outliers and normality, continuous data were summarized using means and standard deviations. Categorical variables were summarized using tables of frequencies and counts. Mean levels of 25-OHD for mothers and newborns were compared across field centers using ANOVA. Linear regression was used to evaluate associations of primary interest. Unadjusted regression models for maternal 25-OHD and maternal BMI, insulin sensitivity, fasting plasma glucose, and GDM were initially examined. Models were then adjusted for maternal age and field center, with additional adjustment for maternal BMI in models for insulin sensitivity, fasting glucose and GDM. For analyses of newborn 25-OHD, unadjusted regression models were initially examined with maternal BMI, birth weight and neonatal adiposity as primary predictors of interest. Adjustments for maternal 25-OHD, birth season, maternal age and field center were then included for all three models, with additional adjustment for sex of the neonate and maternal BMI for the models examining birth weight and neonatal adiposity. Season of birth was dichotomized as winter (November, December, January, February, March, April) or summer (May, June, July, August, September, October) for analysis. All analyses were performed with SAS 9.4 (Cary, NC) and R 3.0.2 (Redmond, WA).

## Results

Maternal and neonatal characteristics of the 360 pairs studied are displayed in Table 1. The mean maternal BMI was 28.5 kg/m^2^ at the HAPO study visit (27.8 ± 1.4 weeks gestation) and 60.0% of women had normal weight BMI, 20.8% were overweight, and 19.2% were obese. Based on fasting, 1-hr and 2-hr plasma glucose levels from the 75-g OGTT, 16.4% of the women in this cohort would be classified as having gestational diabetes mellitus according to the International Association of Diabetes and Pregnancy Study Groups (IADPSG) Consensus Panel [23].

**Table 1:** Maternal and Neonatal Characteristics.

	Mean (SD) or N (%)
**Maternal Characteristics**	N = 360
Age (years)	32.5 (4.6)
Gestational Age at OGTT and BMI measurement (wks)	27.8 (1.4)
BMI (kg/m^2^)	28.5 (5.3)
Normal weight (<28.5)	216 (60.0%)
Overweight (28.5–33)	75 (20.8%)
Obese (>33)	69 (19.2%)
Serum 25-OHD (ng/ml)	37.2 (11.2)
Fasting Plasma Glucose (mg/dl)	82.6 (6.6)
Insulin Sensitivity	3.7 (1.4)
Gestational Diabetes Mellitus^	59 (16.4%)
Field Center	
Chicago, IL	112 (31.1%)
Cleveland, OH	118 (32.8%)
Toronto, ON	130 (36.1%)
**Neonatal Characteristics**	N = 360
Gestational Age (wks)	40.1 (0.7)
Birth Weight (g)	3582.3 (429.2)
Adiposity (% fat)	12.3 (3.3)
Cord blood 25-OHD (ng/ml)	23.4 (9.2)
Birth season	
Winter	181 (50.3%)
Summer	179 (49.7%)

^International Association of Diabetes and Pregnancy Study Groups Consensus Panel [23]

**Fig 1:**
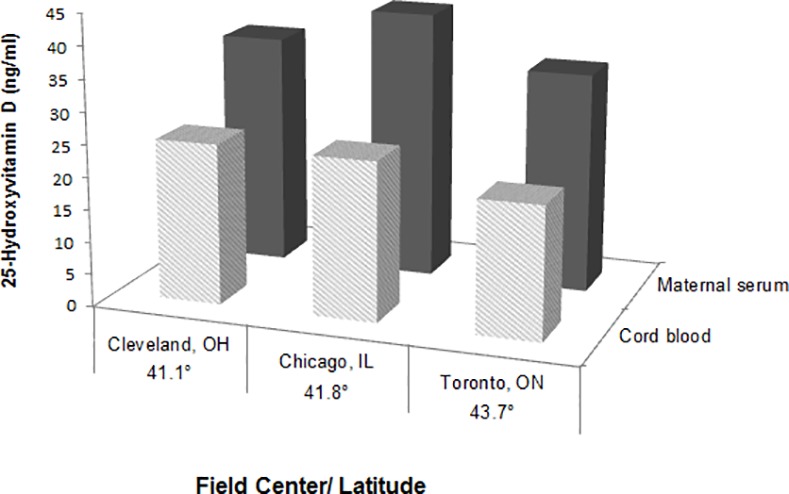
Vitamin D levels by field center. Bars display mean levels +/- sd, solid gray for maternal serum and hatched light grey for cord blood. Cleveland, OH: n = 118; Maternal 25-OHD = 36.1 ± 10.6, cord blood = 25.0 ± 10.0 ng/ml; Chicago, IL: n = 112; Maternal 25-OHD = 41.7 ± 10.7, cord blood = 24.7 ± 9.0 ng/ml; Toronto, ON: n = 130; Maternal 25-OHD = 34.1 ± 11.0, cord blood = 20.6 ± 8.0 ng/ml. Field center levels of 25-hydroxyvitamin D were significantly different for both maternal and cord blood, (ANOVA p< 0.001).

Maternal and cord blood mean levels of 25-OHD are displayed in Fig 1 according to field center with the corresponding latitude. Significant differences of 25-OHD levels among the field centers were found for both maternal levels (ANOVA p<0.001) and cord blood levels (ANOVA p<0.001). Women and neonates from Toronto, the center at the highest latitude, had the lowest 25-OHD levels compared to Cleveland and Chicago. The mean maternal 25-OHD levels across all field centers were > 30 ng/ml.

Linear regression models were used to explore associations between maternal serum 25-OHD levels and maternal BMI, fasting plasma glucose, insulin sensitivity and presence of GDM (Table 2). In univariate analyses, maternal BMI and insulin sensitivity were significantly associated with maternal serum 25-OHD levels. In a model adjusted for maternal age and field center, maternal serum 25-OHD was lower by 0.40 ng/ml for BMI higher by 1 kg/m^2^, (p<0.001). Maternal fasting plasma glucose and GDM presence were not significantly associated with maternal serum 25-OHD level in either unadjusted or adjusted models. While insulin sensitivity was significantly associated with serum 25-OHD level in the univariate model (p = 0.005), this relationship was no longer significant in a model adjusted for maternal age, BMI and field center. Additional adjustment for season at the time of OGTT and maternal blood collection in the model for maternal 25-OHD level did not appreciably change the results (data not shown).

**Table 2:** Linear regression analysis of the relationship between maternal serum 25-OHD at OGTT and maternal factors.

	Univariate model		Adjusted Model^	
	Beta estimate (95% CI)	p value	Beta estimate (95% CI)	p value
BMI (kg/m^2^) at OGTT (27.8 wks)	-0.48 (-0.71, -0.25)	<0.001	-0.40 (-0.62, -0.17)	<0.001
Fasting plasma glucose (mg/dl)	-0.02 (-0.22, 0.17)	0.83	0.05 (-0.14, 0.25)	0.59
Insulin sensitivity	1.29 (0.40, 2.19)	0.005	0.40 (-0.60, 1.39)	0.43
GDM (yes v. no)	0.18 (-3.20, 3.56)	0.92	0.74 (-2.57, 4.04)	0.66

^All models were adjusted for maternal age and field center; fasting plasma glucose, insulin sensitivity and GDM models were additionally adjusted for maternal BMI.

**Fig 2:**
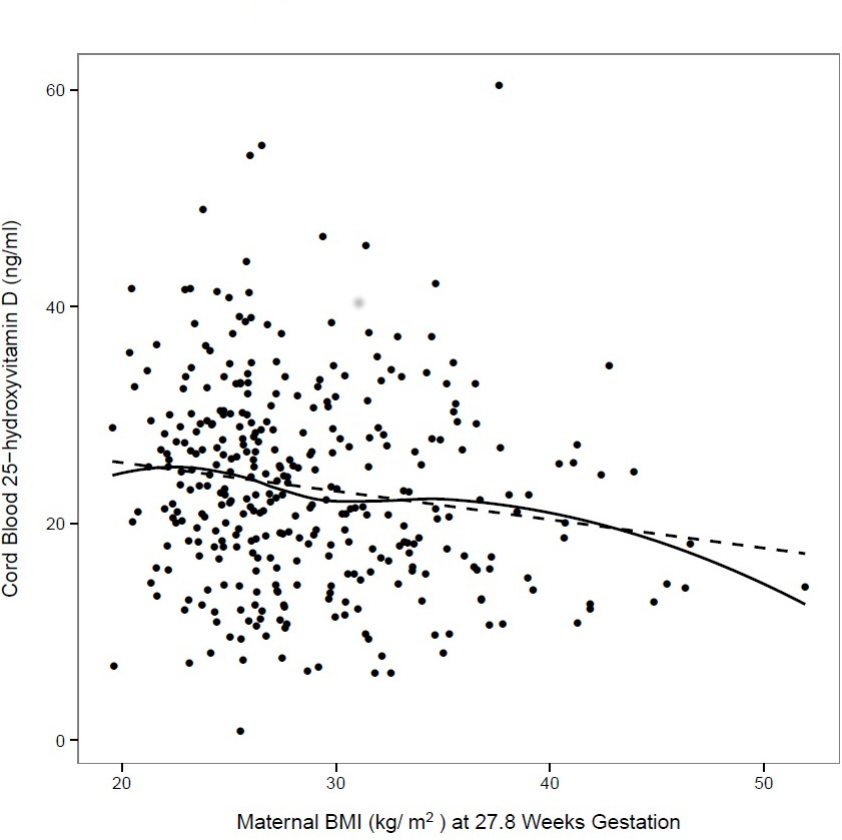
Maternal BMI versus Cord Blood 25-OHD Levels. Scatterplot of cord blood 25-hydroxyvitamin D versus maternal BMI measured at OGTT, N = 360. Both the regression line (dotted line, β estimate = -0.26) and loess curve demonstrate the inverse association between these two variables.

The relationship between maternal BMI and cord blood 25-OHD level is displayed as a scatterplot in Fig 2 with both the estimated regression line and a loess curve confirming the inverse association. In univariate analysis, cord blood 25-OHD is lower by 0.26 ng/ml for maternal BMI higher by 1 kg/m^2^ (p<0.004). However, in the model adjusted for maternal age, field center, birth season and maternal serum 25-OHD, the association of cord blood 25-OHD and maternal BMI is attenuated with maternal serum 25-OHD and birth season demonstrating associations in the expected directions (Table 3). Neither birth weight nor neonatal adiposity was significantly associated with cord blood 25-OHD levels in unadjusted or adjusted models. Cord blood levels of 25-OHD were not different in the offspring of mothers with GDM compared to women without GDM in regression analyses (data not shown).

**Table 3:** Linear regression analysis of factors associated with cord blood 25-OHD levels.

	Univariate regression	
	Beta estimate (95% CI)	p value
Maternal BMI (kg/m^2^)	-0.26 (-0.44,-0.08)	0.004
	Adjusted Model for Maternal BMI^	
	Beta estimate (95% CI)	p value
Maternal BMI (kg/m^2^)	-0.07 (-0.22, 0.08)	0.36
Maternal serum 25-OHD (ng/ml)	0.48 (0.41, 0.55)	<0.001
Birth season (winter vs. summer)	5.97 (4.45, 7.48)	<0.001
	Adjusted Model for Neonatal Measures*	
	Beta estimate (95% CI)	p value
Birth weight (g)	-0.0003 (-0.0022, 0.0016)	0.74
Neonatal Adiposity (% fat)	-0.06 (-0.31, 0.19)	0.64

^The adjusted model for cord blood 25-OHD and maternal BMI included adjustment for maternal serum 25-OHD and birth season, as well as maternal age and field center.

*Birth weight and neonatal adiposity models included adjustment for sex of the neonate, maternal BMI, maternal serum 25-OHD, birth season, maternal age and field center.

## Discussion

The results of this study provide considerable evidence that BMI in pregnancy influences maternal 25-OHD levels. Higher maternal BMI during pregnancy was associated with lower maternal 25-OHD levels when measured at approximately 28 weeks gestation; the higher the BMI, the lower the 25-OHD level. The consequences of lower 25-OHD among women with higher BMI suggests that lower 25-OHD levels may be directly transmitted to their full term neonates, since the higher the maternal BMI, the lower the cord blood 25-OHD level in our univariate analysis. While this relationship between maternal BMI and cord blood 25-OHD was not significant in the model adjusted for maternal 25-OHD levels, this finding may be attributed to the strong association of maternal 25-OHD on cord blood 25-OHD levels. Thus, the effect of maternal BMI on cord blood 25-OHD levels may be largely explained by its association with circulating maternal 25-OHD levels.

As we expected, cord blood levels of vitamin D were found to be strongly associated with birth season. Summer month of birth is presumed to be associated with higher maternal sun exposure preceding the neonate’s birth [24]. This analysis indicates that neonates born in summer months had, on average, 6 ng/ml higher cord blood levels of 25-OHD compared to neonates born in winter months. If we allow that 100 IU daily raises 25-OHD by 1 ng/ml, this would translate to the need for an additional 600 IU of supplementation for mothers expecting in winter months to raise their neonate’s cord blood to summer levels [25]. However, caution for too much vitamin D supplementation should be raised as one study determined higher concentrations of neonatal 25-OHD are possibly associated with an elevated risk of becoming overweight in adult life [26].

The results of this study indicate that mothers with higher BMI are at risk for lower vitamin D levels, and consequently, lower vitamin D levels in their neonates. As addressed previously by our group [12] and others [27], obese women may require larger amounts of vitamin D supplementation compared to normal weight women to provide their neonates with sufficient levels of vitamin D. Yet the definition of neonatal vitamin D sufficiency has not been determined.

The implications of neonatal 25-OHD levels and later health outcomes is an emerging area of interest. A few studies have documented adverse health outcomes in children born to mothers with vitamin D deficiency such as increased adiposity [10], less muscle mass [28], reduced bone mineral density [29] and increased risk of food allergy [11]. There are still insufficient data on whether increased 25-OHD supplementation during pregnancy is warranted [30], as supplemental trials have failed to show a significant impact on maternal and offspring health [31]. Thus, well-designed, interventional studies, with both neonatal and longitudinal follow up outcomes, are necessary in order to provide improved recommendations for vitamin D supplementation in pregnancy.

In this study, neither the presence of GDM [23] nor fasting glucose levels were associated with maternal 25-OHD levels. These results are in contrast to other reports suggesting that vitamin D levels influence the risk of GDM [15, 32]. These reports either used a different diagnostic test of GDM, a different methodology of measuring 25-OHD, or measured maternal 25-OHD at a different pregnancy time point, which may explain these different results. McLeod and colleagues [33], who also conducted an ancillary study on HAPO participants, found a weak, yet significant association between 25-OHD and fasting glucose in an Australian vitamin D replete population. Similar to our results, 25-OHD levels among this Australian HAPO cohort were not different in the women with or without GDM.

Interestingly, insulin sensitivity was significantly associated with maternal serum vitamin D level in univariate analysis. Insulin sensitivity is a measure of how sensitive an individual is to insulin effects. Poor insulin sensitivity indicates insulin resistance and is strongly related to obesity. Thus, similar to the non-significant findings of the maternal fasting glucose model, it was not surprising that insulin sensitivity was no longer significant in a model adjusted for maternal BMI.

While higher BMI in this group of women was associated with lower absolute 25-OHD levels, this cohort of pregnant women had, on average, sufficient levels of 25-OHD according to both the Institute of Medicine and Endocrine Society guidelines [34, 35]. As HAPO participants, this population of women was known to have had optimal prenatal care and thus we can assume that prenatal vitamin use was high. However, vitamin D levels at the start of pregnancy among these women are not known. Furthermore, supplementation of vitamin D during pregnancy confounds the outcomes. Whether the vitamin D amount of 400–600 IU of D_2_ or D_3_ in most prenatal vitamin formulations is sufficient to maintain levels > 30 ng/ml, especially among obese women, remains unknown.

Contrary to our previous report [12], cord blood 25-OHD levels were not associated with neonatal size. The current study was designed to remove potential confounders of cord blood 25-OHD levels, including maternal race and gestational age of the neonate, and samples were selected with even distribution for season of birth. Carefully accounting for these variables supports the observation in this study that cord blood 25-OHD is not associated with birth weight or neonatal adiposity.

A major strength of this planned study was its design to reduce potential confounders of associations between maternal and cord blood vitamin D levels and maternal BMI. Accurate maternal and neonatal measurements, obtained by trained research personnel, was another strength of this study. Additionally, the valuable information on maternal glucose tolerance using fasting oral glucose tolerance tests and measures of insulin sensitivity, each processed by a central laboratory, allowed for analysis of associations with serum 25-OHD.

Despite these strengths, this study has some limitations that require acknowledgment. Maternal levels of serum 25-OHD were not known at the start of pregnancy and only a single measurement of maternal 25-OHD was obtained during pregnancy. The results are not necessarily applicable to the general population as only white women were studied, and it is well established that vitamin D levels are lower among people with darker skin complexions [36, 37]. Information on prenatal vitamin use or additional vitamin D supplementation within this cohort was not collected.

In conclusion, we determined that maternal levels of 25-OHD are related to maternal BMI. Our results also suggest that interpretation of neonatal 25-OHD measurements may need to incorporate specific maternal factors in addition to season of birth and latitude.

## Supporting Information

S1 Table Dataset. Data sheet.
(XLSX)

S2 Table Data Dictionary. Data sheet dictionary of subheadings.
(XLSX)
