# Correction: Maternal BMI Associations with Maternal and Cord Blood Vitamin D Levels in a North American Subset of Hyperglycemia and Adverse Pregnancy Outcome (HAPO) Study Participants

**DOI:** 10.1371/journal.pone.0153339

**Published:** 2016-04-05

**Authors:** The PLOS ONE Staff

The author byline is incorrect. The words “on behalf of the” were erroneously omitted during the typesetting process. Please view the correct author list and affiliations here:

Jami L. Josefson^1,2^*, Anna Reisetter^3^, Denise M. Scholtens^3^, Heather E. Price^4^, Boyd E. Metzger^5^, Craig B. Langman^2,4^, on behalf of the HAPO Study Cooperative Research Group^¶^

1 Ann & Robert H. Lurie Children’s Hospital of Chicago, Division of Endocrinology, Chicago, Illinois, United States of America, 2 Department of Pediatrics, Northwestern University Feinberg School of Medicine, Chicago, Illinois, United States of America, 3 Department of Preventive Medicine, Northwestern University Feinberg School of Medicine, Chicago, Illinois, United States of America, 4 Ann & Robert H. Lurie Children’s Hospital of Chicago, Division of Kidney Diseases, Chicago, Illinois, United States of America, 5 Department of Medicine-Endocrinology, Northwestern University Feinberg School of Medicine, Chicago, Illinois, United States of America

¶ Members of the HAPO Study Cooperative Research Group are listed in the Acknowledgments.

The publisher apologizes for the error.
